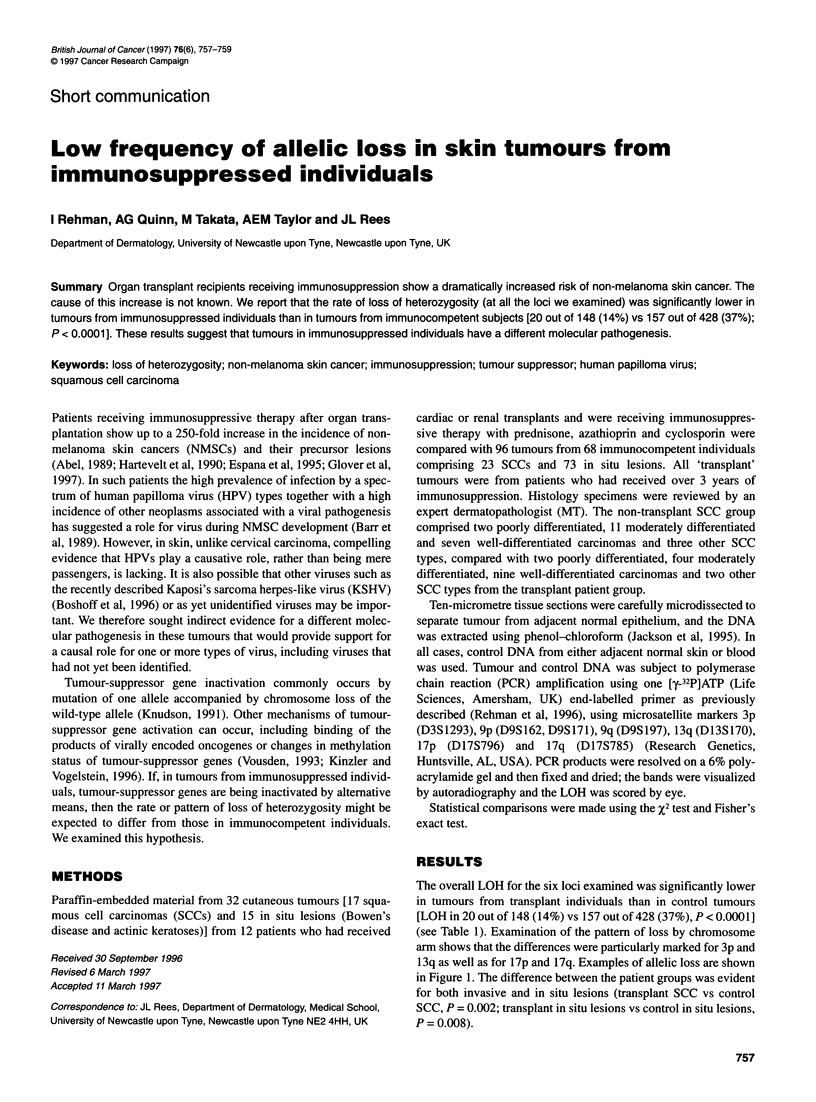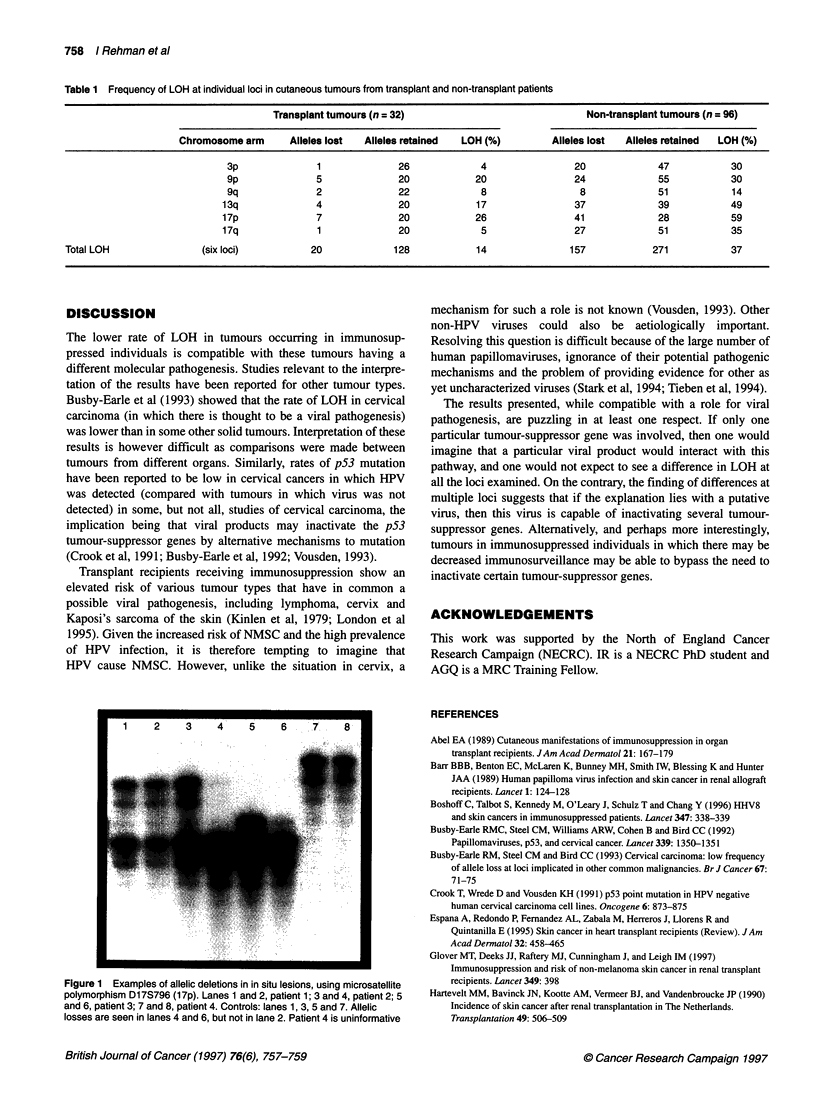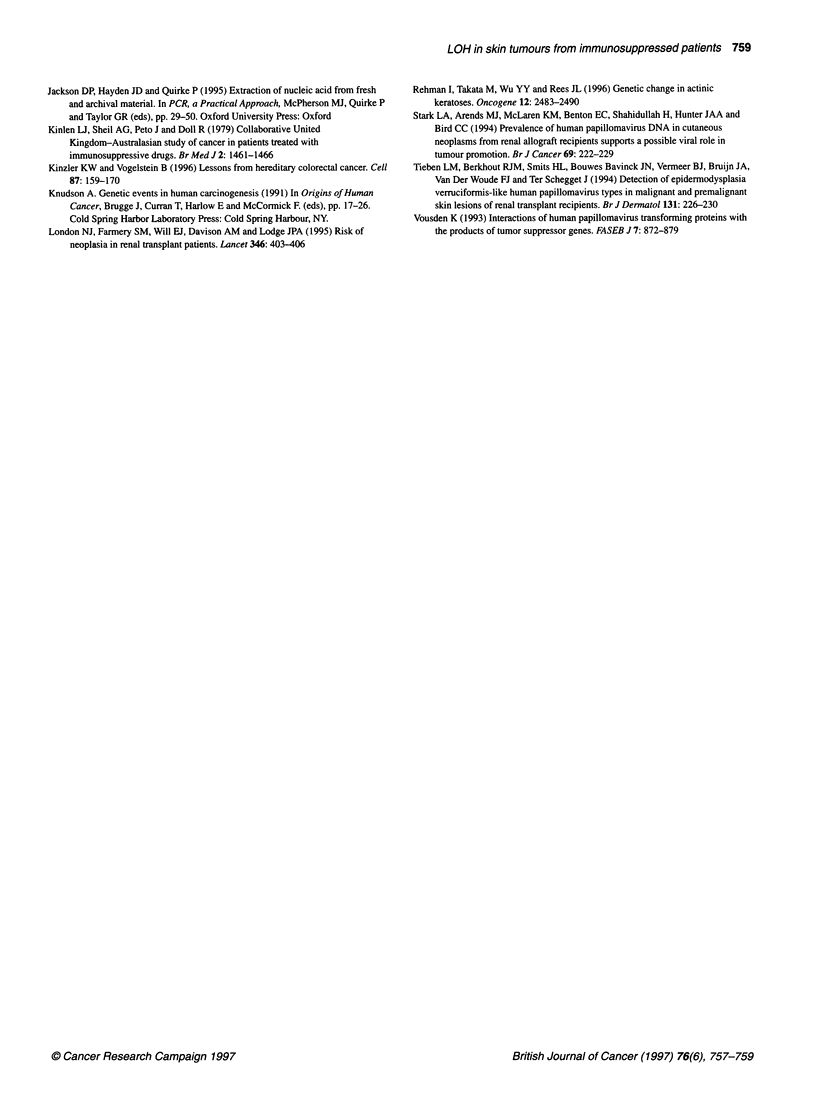# Low frequency of allelic loss in skin tumours from immunosuppressed individuals.

**DOI:** 10.1038/bjc.1997.457

**Published:** 1997

**Authors:** I. Rehman, A. G. Quinn, M. Takata, A. E. Taylor, J. L. Rees

**Affiliations:** Department of Dermatology, University of Newcastle upon Tyne, UK.

## Abstract

**Images:**


*British Journal of Cancer* (1997) **76**(6), 757–759
© 1997 Cancer Research Campaign

Short communication

### Low frequency of allelic loss in skin tumours from immunosuppressed individuals

**I Rehman, AG Quinn, M Takata, AEM Taylor and JL Rees**

Department of Dermatology, University of Newcastle upon Tyne, Newcastle upon Tyne, UK

**Summary** Organ transplant recipients receiving immunosuppression show a dramatically increased risk of non-melanoma skin cancer. The cause of this increase is not known. We report that the rate of loss of heterozygosity (at all the loci we examined) was significantly lower in tumours from immunosuppressed individuals than in tumours from immunocompetent subjects [20 out of 148 (14%) vs 157 out of 428 (37%); $P < 0.0001$]. These results suggest that tumours in immunosuppressed individuals have a different molecular pathogenesis.

**Keywords:** loss of heterozygosity; non-melanoma skin cancer; immunosuppression; tumour suppressor; human papilloma virus; squamous cell carcinoma

Patients receiving immunosuppressive therapy after organ transplantation show up to a 250-fold increase in the incidence of non-melanoma skin cancers (NMSCs) and their precursor lesions (Abel, 1989; Hartevelt et al, 1990; Espana et al, 1995; Glover et al, 1997). In such patients the high prevalence of infection by a spectrum of human papilloma virus (HPV) types together with a high incidence of other neoplasms associated with a viral pathogenesis has suggested a role for virus during NMSC development (Barr et al, 1989). However, in skin, unlike cervical carcinoma, compelling evidence that HPVs play a causative role, rather than being mere passengers, is lacking. It is also possible that other viruses such as the recently described Kaposi's sarcoma herpes-like virus (KSHV) (Boshoff et al, 1996) or as yet unidentified viruses may be important. We therefore sought indirect evidence for a different molecular pathogenesis in these tumours that would provide support for a causal role for one or more types of virus, including viruses that had not yet been identified.

Tumour-suppressor gene inactivation commonly occurs by mutation of one allele accompanied by chromosome loss of the wild-type allele (Knudson, 1991). Other mechanisms of tumour-suppressor gene activation can occur, including binding of the products of virally encoded oncogenes or changes in methylation status of tumour-suppressor genes (Vousden, 1993; Kinzler and Vogelstein, 1996). If, in tumours from immunosuppressed individuals, tumour-suppressor genes are being inactivated by alternative means, then the rate or pattern of loss of heterozygosity might be expected to differ from those in immunocompetent individuals. We examined this hypothesis.

### METHODS

Paraffin-embedded material from 32 cutaneous tumours [17 squamous cell carcinomas (SCCs) and 15 in situ lesions (Bowen's disease and actinic keratoses)] from 12 patients who had received cardiac or renal transplants and were receiving immunosuppressive therapy with prednisone, azathioprin and cyclosporin were compared with 96 tumours from 68 immunocompetent individuals comprising 23 SCCs and 73 in situ lesions. All 'transplant' tumours were from patients who had received over 3 years of immunosuppression. Histology specimens were reviewed by an expert dermatopathologist (MT). The non-transplant SCC group comprised two poorly differentiated, 11 moderately differentiated and seven well-differentiated carcinomas and three other SCC types, compared with two poorly differentiated, four moderately differentiated, nine well-differentiated carcinomas and two other SCC types from the transplant patient group.

Ten-micrometre tissue sections were carefully microdissected to separate tumour from adjacent normal epithelium, and the DNA was extracted using phenol–chloroform (Jackson et al, 1995). In all cases, control DNA from either adjacent normal skin or blood was used. Tumour and control DNA was subject to polymerase chain reaction (PCR) amplification using one [$\gamma$-$^{32}$P]ATP (Life Sciences, Amersham, UK) end-labelled primer as previously described (Rehman et al, 1996), using microsatellite markers 3p (D3S1293), 9p (D9S162, D9S171), 9q (D9S197), 13q (D13S170), 17p (D17S796) and 17q (D17S785) (Research Genetics, Huntsville, AL, USA). PCR products were resolved on a 6% polyacrylamide gel and then fixed and dried; the bands were visualized by autoradiography and the LOH was scored by eye.

Statistical comparisons were made using the $\chi^2$ test and Fisher's exact test.

### RESULTS

The overall LOH for the six loci examined was significantly lower in tumours from transplant individuals than in control tumours [LOH in 20 out of 148 (14%) vs 157 out of 428 (37%), $P < 0.0001$] (see Table 1). Examination of the pattern of loss by chromosome arm shows that the differences were particularly marked for 3p and 13q as well as for 17p and 17q. Examples of allelic loss are shown in Figure 1. The difference between the patient groups was evident for both invasive and in situ lesions (transplant SCC vs control SCC, $P = 0.002$; transplant in situ lesions vs control in situ lesions, $P = 0.008$).

*Received 30 September 1996*
*Revised 6 March 1997*
*Accepted 11 March 1997*

*Correspondence to:* JL Rees, Department of Dermatology, Medical School, University of Newcastle upon Tyne, Newcastle upon Tyne NE2 4HH, UK

**Table 1** Frequency of LOH at individual loci in cutaneous tumours from transplant and non-transplant patients

| | Transplant tumours (*n* = 32) | | | | Non-transplant tumours (*n* = 96) | | |
|---|---|---|---|---|---|---|---|
| | Chromosome arm | Alleles lost | Alleles retained | LOH (%) | Alleles lost | Alleles retained | LOH (%) |
| | 3p | 1 | 26 | 4 | 20 | 47 | 30 |
| | 9p | 5 | 20 | 20 | 24 | 55 | 30 |
| | 9q | 2 | 22 | 8 | 8 | 51 | 14 |
| | 13q | 4 | 20 | 17 | 37 | 39 | 49 |
| | 17p | 7 | 20 | 26 | 41 | 28 | 59 |
| | 17q | 1 | 20 | 5 | 27 | 51 | 35 |
| Total LOH | (six loci) | 20 | 128 | 14 | 157 | 271 | 37 |

### DISCUSSION

The lower rate of LOH in tumours occurring in immunosuppressed individuals is compatible with these tumours having a different molecular pathogenesis. Studies relevant to the interpretation of the results have been reported for other tumour types. Busby-Earle et al (1993) showed that the rate of LOH in cervical carcinoma (in which there is thought to be a viral pathogenesis) was lower than in some other solid tumours. Interpretation of these results is however difficult as comparisons were made between tumours from different organs. Similarly, rates of *p53* mutation have been reported to be low in cervical cancers in which HPV was detected (compared with tumours in which virus was not detected) in some, but not all, studies of cervical carcinoma, the implication being that viral products may inactivate the *p53* tumour-suppressor genes by alternative mechanisms to mutation (Crook et al, 1991; Busby-Earle et al, 1992; Vousden, 1993).

Transplant recipients receiving immunosuppression show an elevated risk of various tumour types that have in common a possible viral pathogenesis, including lymphoma, cervix and Kaposi's sarcoma of the skin (Kinlen et al, 1979; London et al 1995). Given the increased risk of NMSC and the high prevalence of HPV infection, it is therefore tempting to imagine that HPV cause NMSC. However, unlike the situation in cervix, a mechanism for such a role is not known (Vousden, 1993). Other non-HPV viruses could also be aetiologically important. Resolving this question is difficult because of the large number of human papillomaviruses, ignorance of their potential pathogenic mechanisms and the problem of providing evidence for other as yet uncharacterized viruses (Stark et al, 1994; Tieben et al, 1994).

The results presented, while compatible with a role for viral pathogenesis, are puzzling in at least one respect. If only one particular tumour-suppressor gene was involved, then one would imagine that a particular viral product would interact with this pathway, and one would not expect to see a difference in LOH at all the loci examined. On the contrary, the finding of differences at multiple loci suggests that if the explanation lies with a putative virus, then this virus is capable of inactivating several tumour-suppressor genes. Alternatively, and perhaps more interestingly, tumours in immunosuppressed individuals in which there may be decreased immunosurveillance may be able to bypass the need to inactivate certain tumour-suppressor genes.

**Figure 1** Examples of allelic deletions in in situ lesions, using microsatellite polymorphism D17S796 (17p). Lanes 1 and 2, patient 1; 3 and 4, patient 2; 5 and 6, patient 3; 7 and 8, patient 4. Controls: lanes 1, 3, 5 and 7. Allelic losses are seen in lanes 4 and 6, but not in lane 2. Patient 4 is uninformative

### ACKNOWLEDGEMENTS

This work was supported by the North of England Cancer Research Campaign (NECRC). IR is a NECRC PhD student and AGQ is a MRC Training Fellow.

### REFERENCES

Abel EA (1989) Cutaneous manifestations of immunosuppression in organ transplant recipients. *J Am Acad Dermatol* **21**: 167–179

Barr BBB, Benton EC, McLaren K, Bunney MH, Smith IW, Blessing K and Hunter JAA (1989) Human papilloma virus infection and skin cancer in renal allograft recipients. *Lancet* **1**: 124–128

Boshoff C, Talbot S, Kennedy M, O'Leary J, Schulz T and Chang Y (1996) HHV8 and skin cancers in immunosuppressed patients. *Lancet* **347**: 338–339

Busby-Earle RMC, Steel CM, Williams ARW, Cohen B and Bird CC (1992) Papillomaviruses, p53, and cervical cancer. *Lancet* **339**: 1350–1351

Busby-Earle RM, Steel CM and Bird CC (1993) Cervical carcinoma: low frequency of allele loss at loci implicated in other common malignancies. *Br J Cancer* **67**: 71–75

Crook T, Wrede D and Vousden KH (1991) p53 point mutation in HPV negative human cervical carcinoma cell lines. *Oncogene* **6**: 873–875

Espana A, Redondo P, Fernandez AL, Zabala M, Herreros J, Llorens R and Quintanilla E (1995) Skin cancer in heart transplant recipients (Review). *J Am Acad Dermatol* **32**: 458–465

Glover MT, Deeks JJ, Raftery MJ, Cunningham J, and Leigh IM (1997) Immunosuppression and risk of non-melanoma skin cancer in renal transplant recipients. *Lancet* **349**: 398

Hartevelt MM, Bavinck JN, Kootte AM, Vermeer BJ, and Vandenbroucke JP (1990) Incidence of skin cancer after renal transplantation in The Netherlands. *Transplantation* **49**: 506–509

© Cancer Research Campaign 1997

Jackson DP, Hayden JD and Quirke P (1995) Extraction of nucleic acid from fresh and archival material. In *PCR, a Practical Approach,* McPherson MJ, Quirke P and Taylor GR (eds), pp. 29–50. Oxford University Press: Oxford

Kinlen LJ, Sheil AG, Peto J and Doll R (1979) Collaborative United Kingdom–Australasian study of cancer in patients treated with immunosuppressive drugs. *Br Med J* **2**: 1461–1466

Kinzler KW and Vogelstein B (1996) Lessons from hereditary colorectal cancer. *Cell* **87**: 159–170

Knudson A. Genetic events in human carcinogenesis (1991) In *Origins of Human Cancer*, Brugge J, Curran T, Harlow E and McCormick F. (eds), pp. 17–26. Cold Spring Harbor Laboratory Press: Cold Spring Harbour, NY.

London NJ, Farmery SM, Will EJ, Davison AM and Lodge JPA (1995) Risk of neoplasia in renal transplant patients. *Lancet* **346**: 403–406

Rehman I, Takata M, Wu YY and Rees JL (1996) Genetic change in actinic keratoses. *Oncogene* **12**: 2483–2490

Stark LA, Arends MJ, McLaren KM, Benton EC, Shahidullah H, Hunter JAA and Bird CC (1994) Prevalence of human papillomavirus DNA in cutaneous neoplasms from renal allograft recipients supports a possible viral role in tumour promotion. *Br J Cancer* **69**: 222–229

Tieben LM, Berkhout RJM, Smits HL, Bouwes Bavinck JN, Vermeer BJ, Bruijn JA, Van Der Woude FJ and Ter Schegget J (1994) Detection of epidermodysplasia verruciformis-like human papillomavirus types in malignant and premalignant skin lesions of renal transplant recipients. *Br J Dermatol* **131**: 226–230

Vousden K (1993) Interactions of human papillomavirus transforming proteins with the products of tumor suppressor genes. *FASEB J* **7**: 872–879

© Cancer Research Campaign 1997